# Supplementary material for: The interactome of intact mitochondria by cross-linking mass spectrometry provides evidence for coexisting respiratory supercomplexes
Source: Mol Cell Proteomics. 2017 Dec 8;17(2):216–32. doi: 10.1074/mcp.RA117.000470 (PMC5795388; doi:10.1074/mcp.RA117.000470)
Supplement: Supplemental Data [file supp_RA117.000470_133922_0_supp_23978_fzffwf.zip › spectra_annotation/mito_spectra_annotation/344-1-25-1-13-1.pdf]

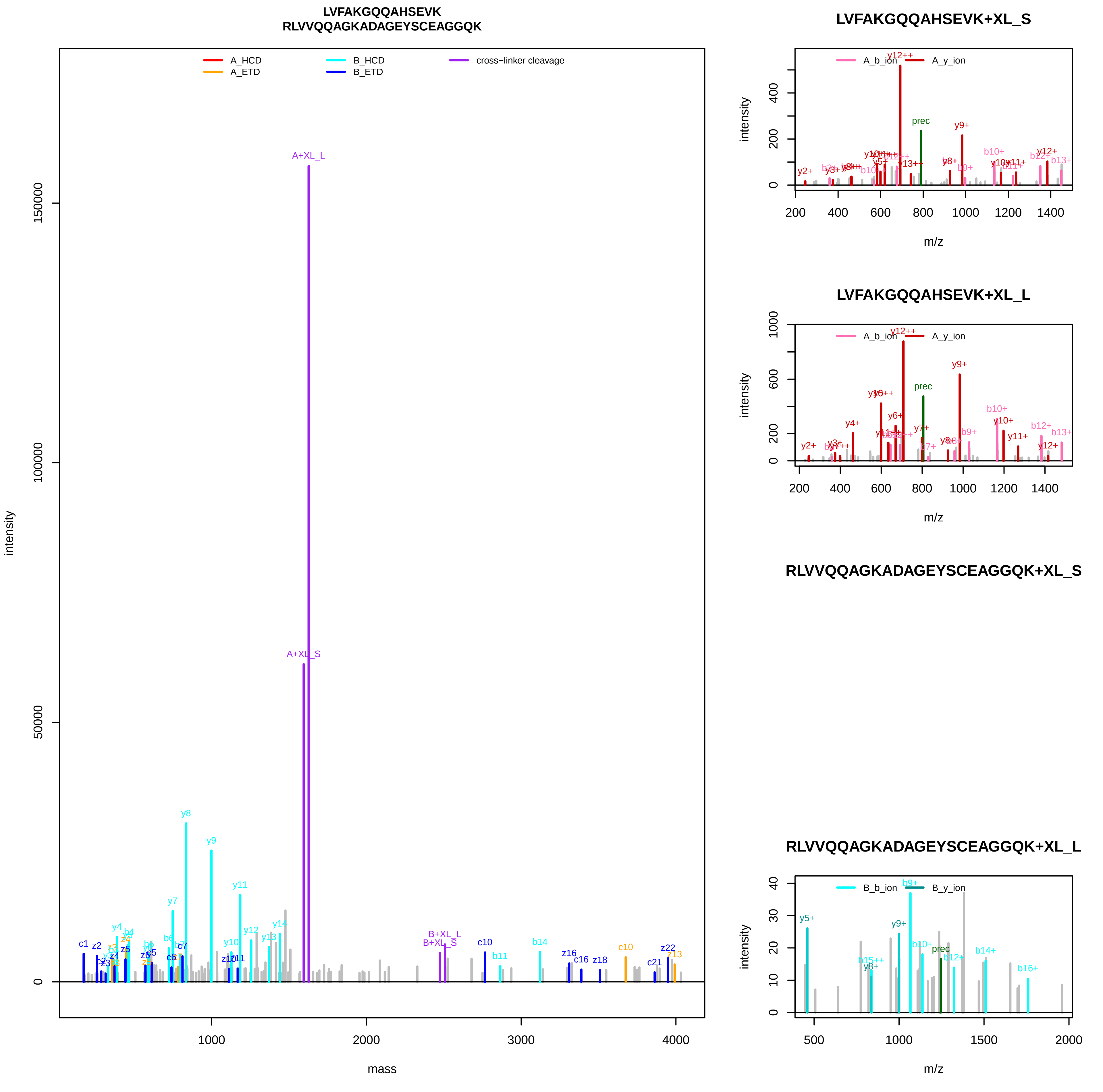
LVFAKGQQAHSEVK
RLVVQQAGKADAGEYSCEAGGQK
A_HCD
A_ETD
B_HCD
B_ETD
cross−linker cleavage
A+XL_L
A+XL_S
B+XL_L
B+XL_S
intensity
mass
0
50000
100000
150000
1000
2000
3000
4000
c1
z2
c5
c6
c7
z5
y4
b4
y7
y8
y9
y10
y11
y12
y13
y14
z12
c11
c10
b11
b14
z16
c16
z18
c10
c21
z22
z13
LVFAKGQQAHSEVK+XL_S
A_b_ion
A_y_ion
y12++
prec
y9+
y8+
y2+
y13++
b10+
b12+
b13+
y12+
m/z
200
400
600
800
1000
1200
1400
0
200
400
LVFAKGQQAHSEVK+XL_L
y9+
y4+
y6+
y7+
y10+
y11+
y12+
b9+
b10+
600
1000
RLVVQQAGKADAGEYSCEAGGQK+XL_S
RLVVQQAGKADAGEYSCEAGGQK+XL_L
B_b_ion
B_y_ion
b9+
y5+
y9+
b10+
y8+
b12+
b14+
b16+
500
1500
2000
10
20
30
40
